# Nut Consumption and Cardiovascular Risk in Older Chinese: The Guangzhou Biobank Cohort Study

**DOI:** 10.1371/journal.pone.0137178

**Published:** 2015-09-02

**Authors:** Yangbo Sun, Chao Qiang Jiang, Kar Keung Cheng, Wei Sen Zhang, Gabriel M. Leung, Tai Hing Lam, C. Mary Schooling

**Affiliations:** 1 School of Public Health, Li Ka Shing Faculty of Medicine, The University of Hong Kong, SAR, China; 2 Guangzhou Number 12 Hospital, Guangzhou, China; 3 Department of Public Health and Epidemiology, University of Birmingham, Birmingham, United Kingdom; 4 School of Public Health, CUNY, New York City, New York, United States of America; Geisel School of Medicine at Dartmouth College, UNITED STATES

## Abstract

**Objectives:**

In Western contexts nut consumption is associated with better health. We examined the associations of nut consumption with cardiovascular disease risk in the non-Western setting of Southern China.

**Methods:**

In the Guangzhou Biobank Cohort Study we used multivariable linear regression to examine the associations of baseline nut (mainly peanuts) consumption (none (n = 6688), <3 portions/week (n = 2596) and ≥3 portions/week (n = 2444)) with follow-up assessment of Framingham cardiovascular disease score (excluding smoking) and its components in older Chinese (≥50 years) (follow-up 57.8%).

**Results:**

Nut consumption was not associated with Framingham score (≥3 portions/week compared to none: 0.02 95% confidence interval (CI) -0.11 to 0.15), systolic blood pressure (-0.66 mmHg 95% CI -1.94, 0.62), diastolic blood pressure (-0.69 mmHg 95% CI -1.44, 0.07), HDL-cholesterol (-0.01 mmol/L 95% CI -0.02, 0.005), LDL-cholesterol (-0.01 mmol/L 95% CI -0.05, 0.02) or fasting glucose (0.04 mmol/L 95% CI -0.02, 0.09), adjusted for baseline values, energy intake, age, sex, phase of recruitment, socio-economic position, lifestyle and baseline health status.

**Conclusions:**

Observations concerning the benefits of nut consumption may be contextually specific, perhaps depending on the type of nut consumed.

## Introduction

Nuts are promoted as part of a healthy diet [1]. Observational studies in Western settings have shown that nut consumption is negatively associated with cardiovascular disease (CVD), diabetes and mortality [2–5]. Long-term randomized controlled trials (RCTs) have shown that the Mediterranean diet, which is rich in nuts, reduces CVD or its risk factors [6, 7]. Some short-term RCTs also showed some protective effects of nut consumption on cardiovascular health, such as lower total and LDL-cholesterol and better glycemic control [8–10]. However, these dietary RCTs are difficult to interpret, because some of the interventions changed more than nut consumption and the type of nuts consumed varied [11]. Moreover, lack of compliance may bias RCTs towards the null.

Nuts are rich in unsaturated fatty acids, minerals (e.g., magnesium and potassium), fibers, plant proteins, and antioxidant vitamins (e.g., vitamin E) [12–14], although the benefits of unsaturated fats and vitamin E have been questioned [15, 16]. The observed associations of nut consumption with cardiovascular health could be the result of beneficial effects of nutrients in nuts, however, it could also be due to residual confounding by socio-economic position (SEP) or other life style factors [17, 18]. Reverse causation is also possible as people might eat more nuts in response to poor health. Evidence from non-western settings with a potentially different confounding structure and diet may help identify whether these observed associations are biologically based or contextually specific results of unmeasured confounding.

To our knowledge no previous study has examined the associations of nut consumption with cardiovascular risk in China. We examined the prospective associations of nut consumption with Framingham score and CVD risk factors in a large study of older adults (50+ years) from the economically developing non-western setting of Guangzhou in Southern China, and whether the associations varied by health status. We also examined whether the associations varied by sex and among men by smoking status.

## Materials and Methods

### Ethics statement

The Guangzhou Medical Ethics Committee of the Chinese Medical Association approved the study and all participants gave written, informed consent before participation.

### Participants

The baseline examination of Guangzhou Biobank Cohort Study (GBCS) was conducted from 2003 to 2008, and the follow-up from 2008 to 2012. Details of GBCS have been reported previously [19]. Briefly, GBCS is a 3-way collaboration between Guangzhou Number 12 Hospital and the Universities of Hong Kong and Birmingham, UK. Recruitment of participants was from “The Guangzhou Health and Happiness Association for the Respectable Elders” (GHHARE), a community social and welfare organization. GHHARE unofficially aligned with the municipal government. Membership is open to Guangzhou permanent residents aged 50 years or above for a nominal fee of 4 CNY (≈50 US cents) per month. GHHARE included about 7% of Guangzhou residents in this age group, with branches in all 10 districts of Guangzhou, the capital city of Guangdong province in southern China. Within sex and age group, the GBCS participants had fairly similar prevalence of chronic diseases, such as diabetes and hypertension, as a nationally representative sample of urban Chinese [19]. The baseline and follow-up examination included an interview concerning lifestyle, family and personal medical history and assessment of weight, height, waist circumference, blood pressure, fasting plasma glucose, lipids and inflammatory markers. Plasma glucose and lipids were measured by Roche COBAS automatic biochemical analyzer.

### Nut consumption

Nut consumption was considered as 25 gram portions per week, obtained from a validated food frequency questionnaire (FFQ) used in phases 1 and 2, but not in phase 3 [19, 20]. The FFQ contained 300 food items, including 5 commonly eaten nuts from Southern China (chestnut, cashew, peanut, walnut and almonds). The participants were asked the usual amount and frequency of each nut consumed in the past week, with a usual serving size specified. Nut consumption was reported as portions per week and categorized into never, <3 portions/week and ≥3 portions/week, based on the frequency of consumption and the usual amount per occasion. The most commonly eaten nut was peanuts, consumed by 36% of the participants. For other types of nut, 10% of participants ever consumed walnuts, 5% ever consumed chestnuts, 2% ever consumed almonds and1% ever consumed cashew nuts.

### Outcomes

The primary outcome was the Framingham score from follow up stage [21] which predicts coronary heart disease (CHD) risk from sex, age, LDL-cholesterol, HDL-cholesterol, blood pressure, smoking and history of diabetes. We removed smoking from the score, so that it was not included, so as to examine the potential biological effect on these risk factors. A higher score predicts higher CHD risk, consistent with the updated risk assessment equations for atherosclerotic CVD in the 2013 American College of Cardiology and the American Heart Association guidelines [22]. The Framingham score over-estimates CVD risk in Chinese populations, but ranks correctly [23]. The secondary outcomes were components of the Framingham score.

### Health status

We constructed a 9-item index to assess health status, similar to one previously used, which was strongly associated with mortality [24], by counting chronic conditions (self-reported heart disease, stroke, diabetes, chronic obstructive pulmonary disease (COPD) and/or asthma, and hypertension), use of health services (regular use of medication and any hospital admission in the last 6 months), cognitive impairment (delayed recall score of 3 or less out of 10), and weight loss of more than 2.5 kg in the last 12 months.

### Statistical analysis

Multivariable linear regression was used to assess the adjusted associations of nut consumption with Framingham score, while multivariable censored linear regression was used to assess associations with blood pressure, lipids and fasting glucose, because people taking medications for hypertension (n = 3871), hyperlipidemia (n = 790) or diabetes (n = 1263) might result in lower blood pressure, LDL-cholesterol and fasting glucose than the true measure and higher HDL-cholesterol. These models censored the outcomes for those on medication at the observed value so that the true measurement for blood pressure, LDL-cholesterol and fasting glucose was assumed to be that observed or higher, while for HDL-cholesterol it was assumed to be that observed or lower. We included potential confounders sequentially. Model 1 adjusted for sex, age (in 5-year age groups), recruitment phase, baseline values of the same risk factor and total energy intake which was calculated from the food frequency questionnaire and dietary composition tables based on McCance and Widdowson [25] and the Chinese Medical Sciences Institute [26]. Model 2 additionally adjusted for life course SEP (father’s occupation, education, longest-held occupation and household income per head) and lifestyle (smoking, alcohol use and physical activity). Model 3 additionally adjusted for health status. Model 4 additionally adjusted for body mass index (BMI) and waist-hip ratio (WHR), considering the unclear role of nut consumption in adiposity.

To assess reverse causality, we examined whether these associations varied by health status, because people in poor health consuming more nuts as a protective measure would generate different associations of nut consumption with CVD risk factors by health status. We assessed differences by health status from the heterogeneity across subgroups and the p-values of the relevant interaction terms in models including interactions with other confounders to avoid confounding by these other interactions. We also similarly examined whether the associations varied by sex, and among men by smoking status.

Information on exposures and potential confounders was missing for less than 5% apart from the longest-held occupation (14.21%) and father’s occupation (42.62%). Missing exposures and potential confounders were predicted based on a flexible additive regression model with predictive mean matching incorporating all exposures, potential confounders and outcomes. We imputed values 10 times and results from the 10 imputations were summarized into single estimated beta-coefficients with confidence intervals and p-values adjusted for the missing data uncertainty [27].

### Sensitivity analysis

Given we previously found SEP was not clearly associated with CVD risk factors among men [28], we did a separate analysis for men, where confounding by socioeconomic factors might be less relevant.

## Results

Of the original 20,305 participants in phases 1 (10389) and 2 (9916) of GBCS, till follow-up 18,569 were still alive, 1446 died and 290 had a status of unknown. Of the 18,569 alive and able to participate in the follow-up, 11,741 participated (63% response), and 11,728 had complete data on nut consumption and energy intake. Compared with those who did not participate in the follow-up, participants in the follow-up were younger, with higher education, a non-manual occupation (of self and father), less likely to be smokers or alcohol users, more physically active, had lower BMI and WHR, and were in better health.

There were more women (8399) than men (3329), and the women were younger (mean age 61.2 (standard deviation (SD) 6.4) than the men (mean age 64.2 (SD 6.2)). Their age ranged from 50 to 95 years, with only 207 participants older than 75 years. Nut consumption (25 gram portions per week) ranged from 0.0 to 70.0, and the median was 0.0 (mean consumption 1.6 (SD 3.0)), with 94% of participants eating less than 7 portions per week.

Greater nut consumption was associated with higher socio-economic position (education, non-manual occupation (of self and father) and higher personal income) for men and women (Table 1). Nut consumption had inconsistent associations with lifestyle and attributes of health, being positively associated with alcohol use, physical activity, and BMI, but was not associated with age, smoking status, WHR or health status.

**Table 1:** Characteristics by Nut Consumption in Older Chinese (3329 men and 8399 women) in Phases 1 and 2 of the Guangzhou Biobank Cohort Study. Abbreviations: IPAQ, International Physical Activity Questionnaire; HEPA, health-enhancing physical activity, i.e., vigorous activity at least 3 days a week that corresponds to a minimum of 1500 metabolic equivalent (MET) minutes per week, or activity 7 days of the week that corresponds to at least 3000 MET minutes per week.

	Nut consumption (25g portions/week)							
	Men				Women			
	0	<3	≥3	^a^ *P* value	0	<3	≥3	^a^ *P* value
N	1956	653	725		4738	1945	1724	
Age group (%)								
50–54	6.8	6.6	7.2		17.0	20.9	18.3	
55–59	20.1	22.5	18.1		28.3	32.3	30.9	
60–64	26.4	25.9	27.7		22.3	20.4	21.9	
65–69	27.4	25.1	28.4		21.4	16.9	19.8	
70–74	17.2	16.7	15.2		9.3	8.5	7.78	
75–79	1.6	2.8	3.0		1.5	0.8	1.2	
≥80	0.5	0.5	0.4	0.37	0.2	0.1	0.1	**<0.001**
Education (%)								
Less than primary school	2.7	1.7	1.7		12.8	9.5	8.2	
Primary school	29.7	23.4	19.5		38.8	33.2	35.3	
Junior middle school	28.2	28.8	32.9		25.2	26.3	30.7	
Senior middle school	24.1	25.0	24.6		18.0	23.7	19.2	
Junior college	8.6	10.7	12.0		3.5	5.0	4.4	
College or above	6.8	10.4	9.4	**<0.001**	1.8	2.4	2.2	**<0.001**
Father’s occupation (%)								
Manual	81.2	81.0	74.3		79.1	76.7	74.6	
Non-manual	18.8	19.0	25.7	**0.01**	20.9	23.3	25.4	**0.01**
Job type (%)								
Manual	57.4	47.8	46.2		78.2	73.8	71.4	
Non-manual	42.6	52.0	53.8	**<0.001**	21.8	26.2	28.6	**<0.001**
Income group (%)								
< 10000 yuan	24.3	20.7	18.8		47.7	38.3	38.6	
10000 to 15000 yuan	44.7	46.1	45.6		41.8	49.7	49.4	
≥ 15000 yuan	31.0	33.2	35.6	**0.02**	10.5	12.0	12.0	**<0.001**
Smoking status (%)								
Never	41.3	48.7	43.3		96.6	97.1	97.0	
Ex-smoker	28.6	25.0	30.2		1.8	1.0	1.4	
Current smoker	30.1	26.3	26.5	**0.01**	1.6	1.9	1.6	0.12
Alcohol use (%)								
Never	64.1	56.9	53.9		91.1	87.8	85.6	
<1/month	12.6	22.2	17.4		5.3	8.1	8.5	
<1/week	4.3	5.4	6.9		1.1	1.5	1.8	
1-4/week	7.8	5.8	9.2		0.9	1.2	1.4	
>5/week	8.6	7.9	10.9		1.2	0.8	1.9	
Ex-drinker	2.7	1.7	1.7	**<0.001**	0.3	0.6	0.8	**<0.001**
Physical activity (IPAQ) (%)								
Inactive	10.1	9.2	5.7		9.9	6.4	4.9	
Minimally active	46.5	44.9	50.2		45.0	48.6	46.0	
HEPA	43.4	45.9	44.1	**0.01**	45.0	45.0	49.1	**<0.001**
Body mass index	23.4	23.5	23.8	**0.02**	23.8	23.9	24.0	**0.04**
Waist hip ratio	0.898	0.900	0.899	0.74	0.855	0.847	0.851	**0.01**
Health status								
Good health	35.0	36.0	31.7		36.1	37.2	36.4	
Poor health	65.0	64.0	68.3	0.19	63.9	62.8	63.6	0.69

^a^ *P* value from chi-square test for categorical variables and from one-way analysis of variants (ANOVA) for continuous variables, 2 sided; bold values indicate *P*<0.05.

Prospectively nut consumption was unrelated to Framingham score and its biological components (systolic blood pressure, diastolic blood, HDL-cholesterol, LDL-cholesterol and fasting glucose) (Table 2). These associations did not vary by health status or sex (p-values≥0.12). Correspondingly, nut consumption was unrelated to Framingham score and its biological components among men only (Table 3), and these associations did not vary by health status or smoking status (p-values≥0.07).

**Table 2:** Adjusted Associations of Nut Consumption with Framingham Risk Score and CVD Risk Factors after Multiple Imputation in Older Chinese in Phases 1 and 2 of the Guangzhou Biobank Cohort Study.

	^b^Model	n	Nut consumption (25g portions/week)					*P*-value for trend	*P*-value for interaction by sex	*P*-value for interaction by health status
			0 (n = 6688)	<3 (n = 2596)		≥3 (n = 2444)				
				^c^coefficient	95% CI	coefficient	95% CI			
^a^Framingham Risk Score	1	11495	reference	0.10	-0.02, 0.23	-0.002	-0.13, 0.13	0.44	0.14	0.23
	2	11495	reference	**0.13**	0.01, 0.26	0.02	-0.11, 0.15			
	3	11495	reference	**0.13**	0.01, 0.26	0.02	-0.11, 0.15			
	4	11495	reference	**0.14**	0.02, 0.26	0.01	-0.12, 0.13			
Systolic blood pressure (mm Hg)	1	11592	reference	-0.59	-1.81, 0.63	-0.62	-1.89, 0.65	0.26	0.69	0.34
	2	11592	reference	-0.64	-1.87, 0.60	-0.64	-1.92, 0.64			
	3	11592	reference	-0.55	-1.78, 0.68	-0.66	-1.94, 0.62			
	4	11592	reference	-0.51	-1.73, 0.72	-0.68	-1.95, 0.60			
Diastolic blood pressure (mm Hg)	1	11595	reference	-0.63	-1.35, 0.09	-0.61	-1.35, 0.14	**0.04**	0.18	0.77
	2	11595	reference	-0.65	-1.37, 0.08	-0.63	-1.38, 0.13			
	3	11595	reference	-0.64	-1.36, 0.09	-0.69	-1.44, 0.07			
	4	11595	reference	-0.69	-1.42, 0.03	**-0.82**	-1.57, -0.07			
HDL-cholesterol (mmol/L)	1	11526	reference	**-0.02**	-0.03, -0.004	-0.01	-0.03, 0.004	0.07	0.51	0.25
	2	11526	reference	**-0.02**	-0.03, -0.004	-0.01	-0.03, 0.004			
	3	11526	reference	**-0.02**	-0.03, -0.004	-0.01	-0.02, 0.005			
	4	11526	reference	**-0.02**	-0.03, -0.004	-0.01	-0.02, 0.01			
LDL-cholesterol (mmol/L)	1	11519	reference	0.004	-0.03, 0.04	-0.02	-0.05, 0.01	0.54	0.12	0.29
	2	11519	reference	0.01	-0.02, 0.04	-0.01	-0.05, 0.02			
	3	11519	reference	0.01	-0.02, 0.04	-0.01	-0.05, 0.02			
	4	11519	reference	0.01	-0.02, 0.04	-0.02	-0.05, 0.02			
Fasting plasma glucose (mmol/L)	1	11685	reference	0.03	-0.02, 0.08	0.04	-0.02, 0.09	0.11	0.36	0.54
	2	11685	reference	0.03	-0.02, 0.09	0.04	-0.01, 0.09			
	3	11685	reference	0.03	-0.02, 0.09	0.04	-0.02, 0.09			
	4	11685	reference	0.04	-0.02, 0.09	0.04	-0.02, 0.09			

^a^Multivariable linear regression was used for Framingham risk score; multivariable censored linear regression was used for blood pressure, cholesterol and glucose.

^b^Model 1 adjusted for same factor at baseline, age, sex, daily energy intake and phase; Model 2 additionally adjusted for SEP (education, father’s occupation, longest-held occupation and personal income) and lifestyle (smoking status, alcohol use and physical activity); Model 3 additionally adjusted for baseline health status: Model 4 additionally adjusted for body mass index and waist-hip ratio.

^c^Coefficient means changes in risk score; bold values indicate *P*<0.05.

**Table 3:** Adjusted Associations of Nut Consumption with Framingham Risk Score and CVD Risk Factors after Multiple Imputation in Older Chinese Men in Phases 1 and 2 of the Guangzhou Biobank Cohort Study.

	^b^Model	n	Nut consumption (25g portions/week)					*P*-value for trend	*P*-value for interaction by health status	*P*-value for interaction bysmoking status
			0 (n = 1952)	<3 (n = 653)		≥3 (n = 724)				
				^c^coefficient	95% CI	coefficient	95% CI			
^a^Framingham Risk Score	1	3262	reference	-0.08	-0.25, 0.09	0.05	-0.12, 0.22	0.48	0.38	0.65
	2	3262	reference	-0.05	-0.22, 0.12	0.08	-0.08, 0.25			
	3	3262	reference	-0.05	-0.22, 0.13	0.08	-0.09, 0.25			
	4	3262	reference	-0.06	-0.23, 0.11	0.06	-0.10, 0.23			
Systolic blood pressure (mm Hg)	1	3282	reference	-1.76	-4.33, 0.82	-0.16	-2.70, 2.37	0.47	0.38	0.99
	2	3282	reference	-2.16	-4.76, 0.43	-0.52	-3.08, 2.05			
	3	3282	reference	-1.97	-4.56, 0.62	-0.54	-3.10, 2.02			
	4	3282	reference	-2.07	-4.64, 0.51	-0.68	-3.23, 1.88			
Diastolic blood pressure (mm Hg)	1	3284	reference	-1.75	-3.59, 0.09	-0.77	-2.59, 1.04	0.13	0.37	0.79
	2	3284	reference	**-1.94**	-3.80, -0.08	-0.99	-2.83, 0.86			
	3	3284	reference	-1.83	-3.69, 0.03	-1.09	-2.93, 0.76			
	4	3284	reference	**-1.89**	-3.75, -0.03	-1.27	-3.11, 0.58			
HDL-cholesterol (mmol/L)	1	3276	reference	-0.001	-0.03, 0.03	-0.01	-0.04, 0.01	0.37	0.56	0.71
	2	3276	reference	-0.01	-0.03, 0.02	-0.01	-0.04, 0.01			
	3	3276	reference	-0.01	-0.03, 0.02	-0.01	-0.04, 0.01			
	4	3276	reference	-0.001	-0.03, 0.02	-0.01	-0.03, 0.02			
LDL-cholesterol (mmol/L)	1	3274	reference	-0.05	-0.11, 0.02	-0.01	-0.07, 0.05	0.81	0.79	0.66
	2	3274	reference	-0.04	-0.10, 0.02	0.002	-0.06, 0.06			
	3	3274	reference	-0.04	-0.10, 0.02	0.002	-0.06, 0.06			
	4	3274	reference	-0.04	-0.10, 0.02	0.003	-0.06, 0.06			
Fasting plasma glucose (mmol/L)	1	3316	reference	-0.03	-0.13, 0.07	0.05	-0.05, 0.15	0.47	0.74	0.07
	2	3316	reference	-0.03	-0.13, 0.07	0.03	-0.05, 0.15			
	3	3316	reference	-0.02	-0.12, 0.08	0.05	-0.05, 0.14			
	4	3316	reference	-0.03	-0.13, 0.07	0.04	-0.05, 0.14			

^a^Multivariable linear regression was used for Framingham risk score; multivariable censored linear regression was used for blood pressure, cholesterol and glucose.

^b^Model 1 adjusted for same factor at baseline, age, daily energy intake and phase; Model 2 additionally adjusted for SEP (education, father’s occupation, longest-held occupation and personal income) and lifestyle (smoking status, alcohol use and physical activity); Model 3 additionally adjusted for baseline health status: Model 4 additionally adjusted for body mass index and waist-hip ratio.

## Discussion

This large prospective study from an under-studied non-western developing population showed no association of nut consumption, mainly peanuts, with Framingham score or its components. The results did not vary by baseline health status or sex, indicating these results were likely not due to changes in response to poor health.

Our results were inconsistent with 6 recent meta-analyses of observational studies from Western countries including prospective studies where nut consumption was found to be associated with lower cardiovascular risk including fewer CVD events and deaths [2–5, 29, 30], and 2 recent meta-analyses of RCTs where nut consumption reduced LDL-cholesterol and glucose [10, 31]. However, our study is consistent with the same meta-analyses of RCTs where nut consumption had no effect on HDL-cholesterol [10, 31], and an RCT where nut consumption was not associated with the risk of myocardial infarction [32]. Some dietary interventions included dietary changes involving more than increased nut consumption and used different types of nuts, which make these dietary RCTs difficult to interpret.

Several explanations for our findings are plausible. Observational studies are vulnerable to residual confounding by SEP, lifestyle and other dietary items. Observational studies in a different population, with justifiable adjustment for confounders, may help verify causality. In China, as in western studies, greater nut consumption was associated with higher SEP and some aspects of a healthier lifestyle [17, 18]. However, our observation that overall nut consumption was not associated with Framingham score or its components is inconsistent with most studies including observational studies from western countries, where nut consumption is usually associated with lower risk of developing CVD. Moreover, in our population for men where there might be less confounding by SEP [28], nut consumption was also not associated with CHD risk factors, indicating confounding by SEP is less likely in our study. Thus differences between the results from our study and western studies may have arisen because our observations are less confounded.

Another possibility is reverse causation, if people in poor health might eat more nuts. In some observational studies in Western countries, the negative associations of nut consumption with CVD risk was attenuated after adjusting for health status suggesting that the observed associations could partially be due to reverse causality [33, 34]. Here we specifically took advantage of our large study to assess reverse causality by examining if associations differed by health status. However, in our study the associations of nut consumption with Framingham score and its components did not vary by health status, indicating reverse causation is unlikely.

We were unable to measure the changes of nut consumption over time, thus it is possible that the associations of nut consumption with cardiovascular risk factors were unobserved if the effect of nut consumption is short-term. The relatively small proportion of large amounts of nut consumption in our population might make it difficult to observe the effect of nuts on cardiovascular risk if high nut consumption is necessary. However, there is no reason to think that nut consumption above a certain threshold is necessary before benefits occur and our large sample makes it possible to detect relatively small effects even within a small range of nut consumption.

Finally, we cannot rule out the possibility that our population were eating the wrong sort of nuts. Peanuts were the type of nuts most commonly consumed, and peanuts are members of the legume family as distinct from tree nuts, such as almonds, walnuts and cashew nuts. Both legumes and tree nuts are part of a healthy diet, although the evidence from RCTs for tree nuts and peanuts is limited and somewhat inconsistent [10, 35], Nevertheless, the benefits of nuts could be restricted to tree nuts.

The strengths of our study included the prospective design, a large sample from an understudied population, whose lifetime experiences were typical of much of the global population from middle and low income countries. Nevertheless, some limitations exist. First, our participants were not a randomly selected, representative sample. However, sample selection should not affect internal associations, unless we missed people with specific combinations of nut consumption and CVD risk. Moreover, when we used inverse probability weighting (IPW) to account for the difference between the original sample and the sample who returned for follow-up, the results were similar (data not shown). Second, we used a single measure of recalled nut consumption. Any non-differential misclassification would likely underestimate the true effects. Systematic recall bias by CVD risk factors is unlikely as participants were unaware of this hypothesis at the time of interview. Third, we were unable to check the influence of nut preparation (e.g., salted, spiced, roasted, or raw) on cardiovascular risk as we lacked data on how nuts were prepared. Finally, we considered the change in CVD risk between baseline and follow-up on the basis that nut consumption at baseline should change risk. However, if baseline nut consumption represents lifelong habits it might be better to examine the association of baseline nut consumption with subsequent CVD risk. The association of baseline nut consumption with Framingham score and its components were similarly null, except nut consumption was positively associated with glucose (Tables C and D in S1 File). However, people who consumed nuts more than 3 portions/week had fasting glucose 0.2 mmol/L higher than people who never consumed nuts on average. Small effect sizes are meaningful at a population level, however an effect size of 0.2 would only represent a very small change in fasting glucose.

It is increasingly apparent that associations between dietary items and cardiovascular disease observed in western settings may reflect correlation rather than causation. Specifically some of the nutrients in nuts, such as unsaturated fats, vitamin E and magnesium have had potentially protective observations with various aspects of cardiovascular health or overall health refuted by RCTs [15, 36, 37]. Thus it is quite plausible that peanuts and/or tree nuts also have no relation with cardiovascular disease, which is only evident in a setting, such as ours, with a different pattern of confounding. In addition, salt may be introduced during the processing of nuts, which might counteract any small benefits of nuts [38].

From a public health perspective, the difficulties of identifying a healthy diet from observed associations are becoming increasingly obvious, consistent with the lack of association of nut consumption with CVD risk in a different setting. Dietary recommendations may need to be based on experimental evidence and should strike a balance between the potential benefits and harms of a certain food, or food pattern.

## Conclusion

Our study is not consistent with western findings that nut consumption can protect against CVD or its risk factors. As with all observational studies these findings should be interpreted cautiously. Given the multi-factorial etiology of CVD, investigation of the underlying factors driving potential health benefits or harms of nuts might facilitate identification of a healthy diet for testing.

## Supporting Information

S1 File Supporting tables. Table A in S1 File Characteristics by Participants and Non-participants during Follow-up in Phases 1 and 2 of the Guangzhou Biobank Cohort Study. Table B in S1 File Framingham Score and Its Components by Nut Consumption in Older Chinese (3329 men and 8399 women) in Phases 1 and 2 of the Guangzhou Biobank Cohort Study. Table C in S1 File Adjusted Associations of Nut Consumption with Framingham Risk Score and CVD Risk Factors after Multiple Imputation in Older Chinese in Phases 1 and 2 of the Guangzhou Biobank Cohort Study (without adjusting for the same factors at baseline). Table D in S1 File Adjusted Associations of Nut Consumption with Framingham Risk Score and CVD Risk Factors after Multiple Imputation in Older Chinese Men in Phases 1 and 2 of the Guangzhou Biobank Cohort Study (without adjusting for the same factors at baseline)
(DOCX)
