# Supplementary material for: Nut Consumption and Cardiovascular Risk in Older Chinese: The Guangzhou Biobank Cohort Study
Source: PLoS One. 2015 Sep 2;10(9):e0137178. doi: 10.1371/journal.pone.0137178 (PMC4558011; doi:10.1371/journal.pone.0137178)
Supplement: S1 File — Table A in S1 File Characteristics by Participants and Non-participants during Follow-up in Phases 1 and 2 of the Guangzhou Biobank Cohort Study. Table B in S1 File Framingham Score and Its Components by Nut Consumption in Older Chinese (3329 men and 8399 women) in Phases 1 and 2 of the Guangzhou Biobank Cohort Study. Table C in S1 File Adjusted Associations of Nut Consumption with Framingham Risk Score and CVD Risk Factors after Multiple Imputation in Older Chinese in Phases 1 and 2 of the Guangzhou Biobank Cohort Study (without adjusting for the same factors at baseline). Table D in S1 File Adjusted Associations of Nut Consumption with Framingham Risk Score and CVD Risk Factors after Multiple Imputation in Older Chinese Men in Phases 1 and 2 of the Guangzhou Biobank Cohort Study (without adjusting for the same factors at baseline) (DOCX) [file pone.0137178.s001.docx]

 Table A Characteristics by Participants and Non-participants during Follow-up in Phases 1 and 2 of the Guangzhou Biobank Cohort Study

|  | Non-participant | | Participant | |  |
| --- | --- | --- | --- | --- | --- |
| N | 8564 |  | 11741 |  | ^a^*P* value |
| Age | 63.7 |  | 62.1 |  | **<0.001** |
| Sex |  |  |  |  |  |
| Men | 29.3 |  | 28.4 |  |  |
| Women | 7.07 |  | 71.6 |  | 0.14 |
| Education (%) |  |  |  |  |  |
| Less than primary school | 13.7 |  | 8.6 |  |  |
| Primary school | 36.3 |  | 33.8 |  |  |
| Junior middle school | 23.9 |  | 27.4 |  |  |
| Senior middle school | 17.2 |  | 20.9 |  |  |
| Junior college | 4.9 |  | 5.7 |  |  |
| College or above | 4.1 |  | 3.7 |  | **<0.001** |
| Father’s occupation (%) |  |  |  |  |  |
| Manual | 80.3 |  | 78.3 |  |  |
| Non-manual | 19.7 |  | 21.7 |  | **0.01** |
| Job type (%) |  |  |  |  |  |
| Manual | 72.1 |  | 69.3 |  |  |
| Non-manual | 27.9 |  | 30.7 |  | **<0.001** |
| Income group (%) |  |  |  |  |  |
| < 10000 yuan | 39.8 |  | 37.6 |  |  |
| 10000 to 15000 yuan | 42.2 |  | 45.2 |  |  |
| ≥ 15000 yuan | 18.0 |  | 17.2 |  | **<0.001** |
| Smoking status (%) |  |  |  |  |  |
| Never | 78.1 |  | 81.6 |  |  |
| Ex-smoker | 10.8 |  | 9.1 |  |  |
| Current smoker | 11.1 |  | 9.3 |  | **<0.001** |
| Alcohol use (%) |  |  |  |  |  |
| Never | 81.3 |  | 81.1 |  |  |
| <1/month | 8.3 |  | 9.1 |  |  |
| <1/week | 1.7 |  | 2.4 |  |  |
| 1-4/week | 3.6 |  | 3.0 |  |  |
| >5/week | 3.9 |  | 3.4 |  |  |
| Ex-drinker | 1.3 |  | 1.0 |  | **<0.001** |
| Physical activity (IPAQ) (%) |  |  |  |  |  |
| Inactive | 8.1 |  | 8.3 |  |  |
| Minimally active | 49.0 |  | 46.3 |  |  |
| HEPA | 42.9 |  | 45.3 |  | **0.001** |
| Body mass index | 23.8 |  | 23.7 |  | **<0.001** |
| Waist hip ratio | 0.87 |  | 0.86 |  | **0.001** |
| Health status |  |  |  |  |  |
| Good health | 30.7 |  | 35.9 |  |  |
| Poor health | 69.3 |  | 64.1 |  | **<0.001** |

Abbreviations: IPAQ, International Physical Activity Questionnaire; HEPA, health-enhancing physical activity, i.e., vigorous activity at least 3 days a week that corresponds to a minimum of 1500 metabolic equivalent (MET) minutes per week, or activity 7 days of the week that corresponds to at least 3000 MET minutes per week.

^a^*P* value from chi-square test for categorical variables and from one-way analysis of variants (ANOVA) for continuous variables, 2 sided; bold values indicate *P*<0.05.

Table B Framingham Score and Its Components by Nut Consumption in Older Chinese (3329 men and 8399 women) in Phases 1 and 2 of the Guangzhou Biobank Cohort Study

|  | Mean and standard deviation | | |  | Nut consumption (25g portions/week) | | | | | | | |
| --- | --- | --- | --- | --- | --- | --- | --- | --- | --- | --- | --- | --- |
|  |  |  |  | 0 (n=6694) | |  | 1-3 (n=2598) | |  | | >3 (n=2449) | |
| CVD risk factor | Men | Women |  | Men | Women | Men | | Women | | Men | | Women |
| Framingham score | 7.5  ±2.5 | 9.4  ±4.0 | | 7.5  ±2.5 | 9.5  ±4.0 | 7.4  ±2.5 | | 9.4  ±4.0 | | 7.6  ±2.5 | | 9.4  ±4.0 |
|  |  |  | |  |  |  | |  | |  | |  |
| Systolic blood pressure (mm Hg) | 135.4  ±26.1 | 132.4  ±46.4 | | 135.3  ±25.5 | 133.3  ±58.9 | 134.4  ±20.9 | | 131.3  ±21.3 | | 136.4  ±31.5 | | 131.4  ±20.7 |
|  |  |  | |  |  |  | |  | |  | |  |
| Diastolic blood pressure (mm Hg) | 75.5  ±17.6 | 72.1  ±12.3 | | 75.8  ±19.7 | 72.2  ±13.7 | 74.7  ±10.7 | | 72.2  ±10.3 | | 75.5  ±16.45 | | 71.9  ±10.12 |
|  |  |  | |  |  |  | |  | |  | |  |
| HDL-cholesterol (mmol/L) | 1.28  ±0.32 | 1.43  ±0.34 | | 1.28  ±0.32 | 1.43  ±0.34 | 1.28  ±0.33 | | 1.42  ±0.33 | | 1.27  ±0.32 | | 1.43  ±0.34 |
|  |  |  | |  |  |  | |  | |  | |  |
| LDL-cholesterol (mmol/L) | 3.27  ±0.81 | 3.51  ±0.84 | | 3.27  ±0.80 | 3.50  ±0.84 | 3.28  ±0.84 | | 3.53  ±0.84 | | 3.27  ±0.81 | | 3.50  ±0.83 |
|  |  |  | |  |  |  | |  | |  | |  |
| Fasting plasma glucose (mmol/L) | 5.57  ±1.46 | 5.61  ±1.57 | | 5.53  ±1.52 | 5.60  ±1.57 | 5.57  ±1.35 | | 5.60  ±1.59 | | 5.67  ±1.40 | | 5.65  ±1.54 |

Table C Adjusted Associations of Nut Consumption with Framingham Risk Score and CVD Risk Factors after Multiple Imputation in Older Chinese in Phases 1 and 2 of the Guangzhou Biobank Cohort Study (without adjusting for the same factors at baseline)

|  | ^b^Model | n | Nut consumption (25g portions/week) | | | | | | | | | *P*-value  for trend | *P*-value for interaction by sex | *P*-value for interaction by health status |
| --- | --- | --- | --- | --- | --- | --- | --- | --- | --- | --- | --- | --- | --- | --- |
|  |  |  | 0 (n=6688) |  | <3 (n=2596) | | |  | ≥3 (n=2444) | | |  |  |  |
|  |  |  |  | | ^c^coefficient | | 95% CI | coefficient | | | 95% CI |  |  |  |
| ^a^Framingham  Risk Score | 1 | 11495 | reference | | 0.10 | -0.05, 0.25 | | 0.02 | | -0.13, 0.17 | | 0.40 | 0.25 | 0.08 |
|  | 2 | 11495 | reference | | 0.14 | -0.01, 0.29 | | 0.05 | | -0.10, 0.20 | |  |  |  |
|  | 3 | 11495 | reference | | 0.13 | -0.01, 0.28 | | 0.03 | | -0.12, 0.18 | |  |  |  |
|  | 4 | 11495 | reference | | **0.14** | 0.01, 0.28 | | 0.01 | | -0.13, 0.15 | |  |  |  |
|  |  |  |  | |  |  | |  | |  | |  |  |  |
| Systolic blood pressure (mm Hg) | 1 | 11592 | reference | | 0.14 | -1.35, 1.64 | | 0.33 | | -1.22, 1.89 | | 0.71 | 0.62 | 0.31 |
|  | 2 | 11592 | reference | | 0.39 | -1.12, 1.89 | | 0.51 | | -1.05, 2.07 | |  |  |  |
|  | 3 | 11592 | reference | | 0.39 | -1.06, 1.83 | | 0.21 | | -1.29, 1.70 | |  |  |  |
|  | 4 | 11592 | reference | | 0.30 | -1.11, 1.71 | | -0.05 | | -1.52, 1.42 | |  |  |  |
|  |  |  |  | |  |  | |  | |  | |  |  |  |
| Diastolic blood pressure (mm Hg) | 1 | 11595 | reference | | -0.48 | -1.33, 0.36 | | -0.54 | | -1.41, 0.34 | | 0.11 | 0.33 | 0.92 |
|  | 2 | 11595 | reference | | -0.40 | -1.25, 0.45 | | -0.52 | | -1.40, 0.37 | |  |  |  |
|  | 3 | 11595 | reference | | -0.41 | -1.24, 0.42 | | -0.67 | | -1.53, 0.20 | |  |  |  |
|  | 4 | 11595 | reference | | -0.55 | -1.36, 0.26 | | -0.95 | | -1.80, -0.11 | |  |  |  |
|  |  |  |  | |  |  | |  | |  | |  |  |  |
| HDL-cholesterol (mmol/L) | 1 | 11526 | reference | | -0.01 | -0.02, 0.01 | | 0.01 | | -0.01, 0.02 | | 0.63 | 0.47 | 0.66 |
|  | 2 | 11526 | reference | | -0.01 | -0.02, 0.01 | | 0.01 | | -0.01, 0.02 | |  |  |  |
|  | 3 | 11526 | reference | | -0.01 | -0.02, 0.01 | | 0.01 | | -0.01, 0.02 | |  |  |  |
|  | 4 | 11526 | reference | | -0.01 | -0.02, 0.01 | | 0.01 | | -0.01, 0.03 | |  |  |  |
|  |  |  |  | |  |  | |  | |  | |  |  |  |
| LDL-cholesterol (mmol/L) | 1 | 11519 | reference | | 0.03 | -0.01, 0.07 | | -0.004 | | -0.04, 0.04 | | 0.86 | 0.42 | 0.06 |
|  | 2 | 11519 | reference | | 0.03 | -0.01, 0.07 | | -0.003 | | -0.04, 0.04 | |  |  |  |
|  | 3 | 11519 | reference | | 0.03 | -0.01, 0.07 | | -0.004 | | -0.05, 0.04 | |  |  |  |
|  | 4 | 11519 | reference | | 0.03 | -0.01, 0.07 | | -0.01 | | -0.05, 0.03 | |  |  |  |
|  |  |  |  | |  |  | |  | |  | |  |  |  |
| Fasting plasma glucose (mmol/L) | 1 | 11685 | reference | | 0.04 | -0.03, 0.12 | | **0.14** | | 0.06, 0.22 | | **0.001** | 0.57 | 0.08 |
|  | 2 | 11685 | reference | | 0.05 | -0.03, 0.13 | | **0.13** | | 0.05, 0.22 | |  |  |  |
|  | 3 | 11685 | reference | | 0.05 | -0.03, 0.13 | | **0.13** | | 0.05, 0.21 | |  |  |  |
|  | 4 | 11685 | reference | | 0.05 | -0.02, 0.13 | | **0.12** | | 0.05, 0.20 | |  |  |  |

^a^Multivariable linear regression was used for Framingham risk score; multivariable censored linear regression was used for blood pressure, cholesterol and glucose.

^b^Model 1 adjusted for age, sex, daily energy intake and phase; Model 2 additionally adjusted for SEP (education, father’s occupation, longest-held occupation and personal income) and lifestyle (smoking status, alcohol use and physical activity); Model 3 additionally adjusted for baseline health status: Model 4 additionally adjusted for body mass index and waist-hip ratio.

^c^Coefficient means changes in risk score; bold values indicate *P*<0.05.

Table D Adjusted Associations of Nut Consumption with Framingham Risk Score and CVD Risk Factors after Multiple Imputation in Older Chinese Men in Phases 1 and 2 of the Guangzhou Biobank Cohort Study (without adjusting for the same factors at baseline)

|  | ^b^Model | n | Nut consumption (25g portions/week) | | | | | | | | | *P*-value  for trend | *P*-value for interaction by health status | *P*-value for interaction by  smoking status |
| --- | --- | --- | --- | --- | --- | --- | --- | --- | --- | --- | --- | --- | --- | --- |
|  |  |  | 0 (n=1952) |  | <3 (n=653) | | |  | ≥3 (n=724) | | |  |  |  |
|  |  |  |  | | ^c^coefficient | | 95% CI | coefficient | | | 95% CI |  |  |  |
| ^a^Framingham  Risk Score | 1 | 3262 | reference | | -0.06 | -0.25, 0.13 | | 0.11 | | -0.08, 0.29 | | 0.43 | 0.80 | 0.76 |
|  | 2 | 3262 | reference | | -0.05 | -0.25, 0.14 | | 0.12 | | -0.07, 0.30 | |  |  |  |
|  | 3 | 3262 | reference | | -0.04 | -0.22, 0.15 | | 0.09 | | -0.09, 0.27 | |  |  |  |
|  | 4 | 3262 | reference | | -0.06 | -0.24, 0.12 | | 0.06 | | -0.11, 0.24 | |  |  |  |
|  |  |  |  | |  |  | |  | |  | |  |  |  |
| Systolic blood pressure (mm Hg) | 1 | 3282 | reference | | -1.10 | -4.15, 1.95 | | 1.39 | | -1.62, 4.39 | | 0.91 | 0.52 | 0.98 |
|  | 2 | 3282 | reference | | -1.47 | -4.53, 1.60 | | 0.88 | | -2.15, 3.92 | |  |  |  |
|  | 3 | 3282 | reference | | -1.08 | -4.03, 1.86 | | 0.47 | | -2.44, 3.38 | |  |  |  |
|  | 4 | 3282 | reference | | -1.39 | -4.28, 1.49 | | -0.03 | | -2.88, 2.83 | |  |  |  |
|  |  |  |  | |  |  | |  | |  | |  |  |  |
| Diastolic blood pressure (mm Hg) | 1 | 3284 | reference | | -1.49 | -3.53, 0.55 | | -0.49 | | -2.50, 1.51 | | 0.19 | 0.28 | 0.60 |
|  | 2 | 3284 | reference | | -1.68 | -3.74, 0.37 | | -0.84 | | -2.87, 1.19 | |  |  |  |
|  | 3 | 3284 | reference | | -1.49 | -3.51, 0.52 | | -1.09 | | -3.09, 0.90 | |  |  |  |
|  | 4 | 3284 | reference | | -1.65 | -3.64, 0.34 | | -1.47 | | -3.45, 0.50 | |  |  |  |
|  |  |  |  | |  |  | |  | |  | |  |  |  |
| HDL-cholesterol (mmol/L) | 1 | 3276 | reference | | 0.01 | -0.02, 0.04 | | 0.003 | | -0.03, 0.03 | | 0.77 | 0.94 | 0.30 |
|  | 2 | 3276 | reference | | 0.01 | -0.02, 0.04 | | 0.001 | | -0.03, 0.03 | |  |  |  |
|  | 3 | 3276 | reference | | 0.01 | -0.02, 0.04 | | 0.003 | | -0.03, 0.03 | |  |  |  |
|  | 4 | 3276 | reference | | 0.01 | -0.02, 0.04 | | 0.01 | | -0.02, 0.04 | |  |  |  |
|  |  |  |  | |  |  | |  | |  | |  |  |  |
| LDL-cholesterol (mmol/L) | 1 | 3274 | reference | | -0.01 | -0.08, 0.06 | | 0.002 | | -0.07, 0.07 | | 0.87 | 0.23 | 0.62 |
|  | 2 | 3274 | reference | | -0.01 | -0.08, 0.07 | | 0.01 | | -0.06, 0.08 | |  |  |  |
|  | 3 | 3274 | reference | | -0.01 | -0.08, 0.07 | | 0.01 | | -0.07, 0.08 | |  |  |  |
|  | 4 | 3274 | reference | | -0.01 | -0.09, 0.06 | | 0.01 | | -0.07, 0.08 | |  |  |  |
|  |  |  |  | |  |  | |  | |  | |  |  |  |
| Fasting plasma glucose (mmol/L) | 1 | 3316 | reference | | 0.06 | -0.08, 0.20 | | **0.22** | | 0.08, 0.35 | | **0.01** | 0.70 | 0.79 |
|  | 2 | 3316 | reference | | 0.04 | -0.10, 0.18 | | **0.20** | | 0.06, 0.34 | |  |  |  |
|  | 3 | 3316 | reference | | 0.05 | -0.09, 0.19 | | **0.19** | | 0.05, 0.33 | |  |  |  |
|  | 4 | 3316 | reference | | 0.04 | -0.10, 0.17 | | **0.18** | | 0.05, 0.32 | |  |  |  |

^a^Multivariable linear regression was used for Framingham risk score; multivariable censored linear regression was used for blood pressure, cholesterol and glucose.

^b^Model 1 adjusted for age, daily energy intake and phase; Model 2 additionally adjusted for SEP (education, father’s occupation, longest-held occupation and personal income) and lifestyle (smoking status, alcohol use and physical activity); Model 3 additionally adjusted for baseline health status: Model 4 additionally adjusted for body mass index and waist-hip ratio.

^c^Coefficient means changes in risk score; bold values indicate *P*<0.05.
